# New scoring schema for finding motifs in DNA Sequences

**DOI:** 10.1186/1471-2105-10-93

**Published:** 2009-03-20

**Authors:** Fatemeh Zare-Mirakabad, Hayedeh Ahrabian, Mehdei Sadeghi, Abbas Nowzari-Dalini, Bahram Goliaei

**Affiliations:** 1 Department of Bioinformatics, Institute of Biochemistry and Biophysics, University of Tehran, Tehran, Iran; 2 Center of Excellence in Biomathematics, School of Mathematics, Statistics, and Computer Science, University of Tehran, Tehran, Iran; 3 National Institute of Genetic Engineering and Biotechnology, Tehran, Iran; 4 School of Computer Science, Institute for Studies in Theoretical Physics and Mathematics (IPM), Tehran, Iran

## Abstract

**Background:**

Pattern discovery in DNA sequences is one of the most fundamental problems in molecular biology with important applications in finding regulatory signals and transcription factor binding sites. An important task in this problem is to search (or predict) known binding sites in a new DNA sequence. For this reason, all subsequences of the given DNA sequence are scored based on an scoring function and the prediction is done by selecting the best score. By assuming no dependency between binding site base positions, most of the available tools for known binding site prediction are designed. Recently Tomovic and Oakeley investigated the statistical basis for either a claim of dependence or independence, to determine whether such a claim is generally true, and they presented a scoring function for binding site prediction based on the dependency between binding site base positions. Our primary objective is to investigate the scoring functions which can be used in known binding site prediction based on the assumption of dependency or independency in binding site base positions.

**Results:**

We propose a new scoring function based on the dependency between all positions in biding site base positions. This scoring function uses joint information content and mutual information as a measure of dependency between positions in transcription factor binding site. Our method for modeling dependencies is simply an extension of position independency methods. We evaluate our new scoring function on the real data sets extracted from JASPAR and TRANSFAC data bases, and compare the obtained results with two other well known scoring functions.

**Conclusion:**

The results demonstrate that the new approach improves known binding site discovery and show that the joint information content and mutual information provide a better and more general criterion to investigate the relationships between positions in the TFBS. Our scoring function is formulated by simple mathematical calculations. By implementing our method on several biological data sets, it can be induced that this method performs better than methods that do not consider dependencies.

## Background

DNA-binding proteins, called transcription factors (TFs), are involved in transcription regulation. These factors bind to specific positions in promoter regions for modulating the expression of genes. The common pattern of the recognition sites of a TF is called a *motif*. We use the term *transcription factor binding site *or *motif instance *to mean the occurrence of the motif with some mutations in promoter regions.

Identifying transcription factor binding sites (TFBSs) in promoter regions is a difficult problem in molecular biology. The main reason for this difficulty is that a single transcription factor might bind to regions which vary greatly in their sequences. Although the binding sites for a particular transcription factor share short similar subsequences, sometimes they are highly degenerated. Such short sequences are expected to randomly occur every few hundred base pairs, and thus finding them is a difficult task. Since experimental procedures to determine the exact binding sites are too expensive and time-consuming, computational methods have been developed in the past two decades for discovering novel motifs and TFBSs in a set of promoter sequences [1,2].

There are two main classes of algorithms for finding regulatory motifs. First, the methods that search for known transcription factor binding sites in a new sequence (known motif prediction). Example tools include ConSite [3], Match [4], Mapper [5], Patser [6] and rVista [7]. Second, the methods that try to detect new motifs within a set on DNA sequences based on sequence homology (unknown motif prediction). Example tools include Gibbs sampler [8], AlignACE [9], MEME [10] and Yeast Motif Finder [11]. Generally, motif finding algorithms in both of above methods have three important elements: a motif model that can capture the similarities of a diverse set of binding sites for the same transcription factor, an objective function defining the ranking of known motif (in the first methods) or potential motifs (in the second methods), and a search strategy for parameterizations of the motif model. The first two elements can be given an abstract representation or modeling, but should probably be designed to utilize and enhance biologically relevant information.

Until now, the most common way for binding sites modeling is to assume that any base in each site occurs independent of others. In this modeling, a motif is represented based on consensus sequences [12], position weight matrices (PWMs) [9,10], matrix profiles [13], sequence logos [14], mismatch strings (MMs) [15,16] (consensus string allowing some mismatches) and IUPAC strings (IUPACs) [11,17] (consensus string with degenerate symbols). Methods based on the assumption of independency between positions are simple with small number of parameters that make them easy to implement. These methods are widely used and often considered as acceptable models for binding-site predictions [18]. However, recent experimental evidence [18,19] has promoted the development of models which incorporate position dependencies. The related methods include Bayesian networks [20], permuted Markov models [21], Markov chain optimization [22], hidden Markov models [5], non-parametric models [23] and generalized weight matrix models [1]. Based on the above discussion, another method for modeling binding sites is presented by Tomovic and Oakeley [24]. In this method, for a given TFBS, dependent and independent positions are considered and in searching for a motif the scoring is calculated based on them. The dependency between positions of a given TFBS are predicted by statistical approach which may be explained by structure of TF-DNA complexes. Methods based on position dependencies usually have better binding site prediction accuracy with lower false positive rates. But these methods require more complicated mathematical tools, with more parameters to estimate, and require more experimental data than typically available ones [5,20,22,23]. On one hand, a more comprehensive model may allow for a better fit to the data. On the other hand, the more complex model may over-fit the data and result in an inferior predictive power.

In this paper, we focus on TFBS modelings and search methods for known motif prediction which find known transcription factor binding sites in a given sequence, and investigate known motif ranking (scoring schema). We study whether TFs show position dependencies in their binding sites or not. We also investigate the use of joint information content and mutual information as a measure of dependency between positions in TFBS. We suggest a statistical approach for testing dependencies, and present a new scoring schema that can be used in search methods for finding known transcription factor binding sites. Our method for modeling dependencies is simply an extension of position independencies methods. This method is formulated by simple mathematical calculations, and as will be shown, the proposed algorithm is very simple and substantially efficient, and can be easily implemented on any data sets. We test our new scoring schema on the real data sets and compare the obtained results with two other well known independent and dependent scoring schemas. Using this comparison we can demonstrate the effectiveness of our proposed method against the independent scoring schema, and our scoring function performs better than methods that do not consider dependencies. It is shown that the joint information content and mutual information provide a better and more general criterion to investigate the relationships between positions in the TFBS. Also by using these two measurements, we can obtain results compatible to the results obtained by dependent scoring schema.

## Methods

As mentioned in previous section, one of the important problems in motif discovery area is finding the known TFBSs in a given DNA sequence or promoter region (known motif prediction). In this section we focus on this problem and at first, some definitions and notations further used in this paper are introduced. Let *N *= {*A*, *C*, *G*, *T*} be the four nucleotide letters' of which DNA sequences are composed. We have the DNA sequence *D *= *d*_1_,..., *d*_*n *_(a promoter region) on *N*, and let us suppose that we have *t *known TFBSs of the length ℓ which are represented by a matrix *B*_*t *× ℓ _for a given TF, and we intend to investigate by *B*, where *D *possess a motif instance or transcription factor binding site corresponding to the given TF. For finding the position of this motif instance in *D*, we first create a position weight matrix *W *of *B*, and then we scan all subsequences *R *= *d*_*i*_,..., *d*_*i*+ℓ-1 _for *i *= 1,..., *n *- ℓ + 1 of *D*, and align position weight matrix *W *with each *R*. All the subsequences which their score are greater than a *cutoff *are reported as motif instances. The creation of position weight matrix *W *from TFBSs and calculating the score of alignment *W *with a subsequence are called scoring schema.

The accuracy of the solution in this search problem depends on how we design the scoring schema, and how the position weight matrix is constructed. In this section we first discuss two existing scoring schemas which are employed for ranking known motifs and predicting TFBSs [24], later a new scoring schema is presented.

### Independent scoring schema

The first scoring schema is a conventional method and is employed in many papers [4,8,11,16,25,26]. In this scoring schema, it is assumed that all positions in a given motif are completely independent. This scoring schema is defined as follows.

Suppose we have a promoter region *D *and a TFBS matrix *B *of some known motifs. Assume that *F*(*b*, *j*) (*b *∈ *N *and 1 ≤ *j *≤ ℓ) shows the occurrences of nucleotide *b *in column *j *of the matrix *B*. Employing this function, a probability *P *is made as follows:



where *a*(*b*) is the smoothing parameter (*a*(*b*) = 0.01). Later, a position weight matrix *W*_4 × ℓ _is made as follows:



where each *p*(*b*) shows the occurrence probability of nucleotide *b *(independent of nucleotides in the other position) in a random sequence (obviously *p*(*b*) = 0.25 for every *b *∈ *N*).

Now, let *R *be a DNA subsequence with the length ℓ of a promoter region *D *(*R *= *r*_1_,..., *r*_ℓ_, and *r*_*i *_∈ *N *for 1 ≤ *i *≤ ℓ). For computing the score of *R*, we align position weight matrix *W *with *R *and calculate *Score*_1_(*R*) as follows:



This score can be normalized as follows:



where *MaxScore*_1 _and *MinScore*_1 _are calculated as follows:



### Dependent scoring schema

The second scoring schema was first introduced in [24]. In this scoring schema, dependency between some positions in a given TFBS is assumed. This method uses a statistical approach to find dependent positions in a set of known TFBSs. Therefore, if the dependent positions of a set of TFBSs are available, then this scoring schema is defined as follows.

Similar to the previous definition, we have a promoter region *D *and *t *binding sites of the length ℓ which are represented by a matrix *B*_*t *× ℓ _for a given TF. Also, assume that *F *([*b*_1_,..., *b*_*m*_], [*j*_1_,..., *j*_*m*_]) shows the occurrences of bases *b*_1_,..., *b*_*m *_(*b*_*i *_∈ *N *for 1 ≤ *i *≤ *m*) in dependent positions *j*_1_,..., *j*_*m *_in the matrix *B *(positions *j*_1_,..., *j*_*m *_are determined by statistical approaches [24]). As an example, *F*([*A*, *C*, *A*, *T *],[3, 4, 8, 11]) represents the number of occurrences of A, C, A, and T in the positions 3, 4, 8, and 11 in a given matrix *B*. It should be noted that the positions *j*_1_,..., *j*_*m *_are dependent and not necessarily consecutive.

The corrected probability for the bases *b*_1_,..., *b*_*m *_in positions *j*_1_,..., *j*_*m *_is defined as:



where *a*(*b*_1_,..., *b*_*m*_) is a smoothing parameter and can be calculated as follows:

*a*(*b*_1_,..., *b*_*m*_) = *a*(*b*_1_) × ... × *a*(*b*_*m*_).

Now, the position weight matrix *W *corresponding to the binding sites is calculated as:



Finally, for a given subsequence *R *= *r*_1_,..., *r*_ℓ _(*r*_*i *_∈ *N *and 1 ≤ *i *≤ ℓ) of *D*, we align position weight matrix *W *with *R *and calculate *Score*_2_(*R*) as follows:



where *k*_1 _is the number of independent positions, *k*_2 _is the number of dependent positions order 2 (nucleotides at positions *j*_*i *_and *j*_*i*+1_) and *k*_*m *_the number of dependent positions order *m *(nucleotides at positions *j*_*i*_, *j*_*i*+1_,..., *j*_*i*+*m*-1_).

The normalized version of *Score*_2_(*R*) can be defined as:



where *MaxScore*_2 _and *MinScore*_2 _can be calculated as follows:



### New scoring schema

In the previous subsections we presented two scoring schemas. In the first, nucleotides in all positions in a given TFBS are considered as independent, but this may not be true in all cases because it is shown that dependency between some positions are important [19,27]. In the second, dependency between some positions in a TFBS are considered, but this model has also two problems: first, calculation of dependency between positions is sophisticated, and second, final score is obtained by summation of all the scorings obtained by each order dependent positions, which are not in the same range.

As mentioned, all positions in TFBSs may be dependent, because the length of TFBSs are short, therefore all positions in TFBS may be involved in the interaction with a factor and dependency between all positions are important. TFBSs are short regions in promoter region that TFs can be bonded to them to provide initial conditions for gene transcription. By mutual comparison of TFBS corresponding to a specific TF, we see that some positions in TFBS are mutated and some other ones are conserved. Since the length of a TFBS is short, therefore it seems that both mutated and conserved positions play an important role in binding of TF and TFBS. During a transcription process, TFBS region constructs structure by hydrogen bonds and this causes the attraction of TF to this region. Thus, with respect to the above feature of this process, it seems that the conserved positions and mutated positions cause this attraction. Also, with respect to that, the average specific free energy of binding to all binding sites play an important role in this attraction, and by considering that this energy is directly related to the information content of the preferred binding sites [26], we use the information content for TFBS scoring.

Similar to the previous subsection, suppose that we have a promoter region *D *and binding site matrix *B*_*t *× ℓ _for a given TF. Employing information theory, we compute the information content (*IC*) of a set of TFBSs which are represented by the matrix *B *with position independency as follows.



where *F *and *p *are computed similar to independent scoring schema. From this formula, we have 0 ≤ *IC *≤ 2ℓ. Now, we assume that positions are mutually dependent, and *F*([*b*_1_, *b*_2_], [*j*_1_, *j*_2_]) shows the number of the occurrence of nucleotides *b*_1 _and *b*_2 _in positions *j*_1 _and *j*_2 _in the given matrix *B*. As an example *P*([*A*, *T *], [3, 8]) represents the probability of the occurrence of the pair A and T in the positions 3 and 8 in a given matrix *B*. Clearly, the number of all two combinations of four nucleotides is equal to 16, and the number of all two combinations of ℓ tuples is equal to ℓ (ℓ - 1)/2. In this case, the joint information content (*JIC*) is computed as:



and for this formula we have 0 ≤ *JIC *≤ 4ℓ.

Obviously, we get more information from *JIC *when the positions are more conserved. Now, the problem is to add up the information of the mutated positions to *JIC *which have not been considered yet. For this reason, we compute the mutual information (MI) as follows:



and from this formula we have 0 ≤ *MI *≤ 2ℓ. The relation of *MI *and *JIC *for each position pairs is as follows. If *MI *= 0 then *JIC *= 4 and consequently *MI *+ *JIC *= 4, if *MI *= 2 then *JIC *= 2 and consequently *MI *+ *JIC *= 4. This condition implies that *JIC *does show less information and by adding up MI we can get more information. Actually *MI *carries meaningful information that can not be discarded. On the other hand, *IC *= 2 means, conservation is low but dependency between positions is high.

With regard to the above discussion, the probability of the bases *b*_1 _and *b*_2 _in positions *j*_1 _and *j*_2 _can be defined as:



where *a*(*b*_1_, *b*_2_) is a smoothing parameter and can be calculated as:

*a*(*b*_1_, *b*_2_) = *a*(*b*_1_) × *a*(*b*_2_).

Now, for our scoring schema, we make a position weight matrix *W*_16 × (ℓ(ℓ-1)/2) _whose each entry shows the number of occurrences of a pair of nucleotides in a pair of positions. This matrix is defined as:



where [*b*_1_, *b*_2_] ∈ (*N *× *N*), 1 ≤ *j*_1_, *j*_2 _≤ ℓ, and *j*_1 _≠ *j*_2_.

Finally, for a given subsequence *R *= *r*_1_,..., *r*_ℓ _(*r*_*i *_∈ *N *and 1 ≤ *i *≤ ℓ) of *D*, we align position weight matrix *W *with *R *and evaluate *Score*_3_(*R*) as follows:



The normalized version of *Score*_3_(*R*) can be defined as:



where *MaxScore*_3 _and *MinScore*_3 _are formulated as follows:



## Results and discussion

In order to determine the distribution of TFs with dependent positions and verify that our scoring schema indeed improves the specificity of known motif discovery, we extract some TFs from two public databases JASPAR [28] and TRANSFAC [29]. For extracting the TFs from JASPAR, we select all TFs from JASPAR database and implant TFBSs from these TFs in some random sequences which are generated by the similar way to [24]. For extracting the motifs from TRANSFAC, we use the benchmark data sets that generated by Sandve et al. [30] and Tompa et al. [31]. Sandve generated three data set versions from TRANSFAC based on the collections of binding site fragments that are ranked according to the optimal level of discrimination. These data sets are called 'algorithm-Markov', 'algorithm-real', and 'model-real'. Tompa also generated three data set versions from TRANSFAC based on the employed background sequences. These data sets are called 'Generic', 'MChain', and 'Real'. Therefore, we have seven data sets (JASPAR, algorithm-Markov, algorithm-real, model-Real, Generic, MChain and Real) that each of them contains some TFs (motifs) where each TF contains some TFBSs (motif instances).

We compare our new scoring schema with the two scoring schemas that were introduced in Section 2 on the above data sets for finding known motif instances. The comparisons are proceeded in two levels: Comparison of sites (site level) and comparison of nucleotides (nucleotide level) regarding the position of motifs in the main sequences. For this reason, we first introduce the following criteria for comparison [31].

1.*nTP *is the number of nucleotide positions in both known sites and the predicted sites.

2. *nFP *is the number of nucleotide positions not in the known sites but in the predicted sites.

3. *nFN *is the number of nucleotide positions in known sites but not in the predicted sites.

4. *nTN *is the number of nucleotide positions in neither known sites nor the predicted sites.

5. *sTP *is the number of known sites overlapped by the predicted sites.

6. *sFP *is the number of predicted sites not overlapped by the known sites.

7. *sFN *is the number of known sites not overlapped by the predicted sites.

A predicted site overlaps a known site if it overlaps by at least 25% of the length of the known site. Clearly, the first four criteria are in the nucleotide level and the last three criteria are in the site level. Regarding the above criteria, eight different measurements for the evaluation of the algorithm are introduced.

1. Nucleotide Performance Coefficient (*nPC*): Following Pevzner and Sze [25], *nPC *is defined in the nucleotide level of the predicted sites and is equal to



As we can see *nPC *≤ 1 and the higher value of *nPC *shows that the known sites and the predicted sites are more similar. Obviously, if the predicted sites were equal to the known sites then *nPC *is equal to one.

2. Nucleotide Correlation Coefficient (*nCC*): Following Burset and Guigo [32], *nCC *is defined in the nucleotide level as



The value of *nCC *varies from -1 (indicating perfect anti-correlation between two known sites and the predicted sites) to +1 (indicating the perfect correlation and match).

3. Nucleotide Specificity (*nSp*): A statistical measure for the correctness prediction of positions of a non-motif sequence and is equal to



This measure is called true negative rate in the nucleotide level. The complement of this value is recognized as Nucleotide Selectivity (*nSl*) or false positive rate, i.e. *nSl *= 1 - *nSp*.

4. Nucleotide Sensitivity (*nSn*): is the fraction of the known site nucleotides that are predicted as motifs and is defined by



This measure is called true positive rate in the nucleotide level.

5. Site Sensitivity (*sSn*): is the fraction of predicted sites that are known as



This measure is also called true positive rate in site level.

6. Nucleotide Positive Prediction (*nPP*): is the fraction of the number of nucleotides in the predicted site similar with the number of nucleotides in the known site and is equal to



7. Site Positive Prediction (*sPP*): is the fraction of the number of predicted sites similar with the known sites as



8. Site Average Performance (*sAP*): is the average of site sensitivity and site positive prediction and is defined by



None of the above measurements, can capture the correlation of the motif prediction algorithms perfectly by themselves. Therefore, in any case, we need a way of summarizing the performance of a given motif finding program over all data sets. For each program, each measurement *M *(one of the above eight measurements), over all data sets, is obtained and the performance of each program on all data sets are compared by the similar methods given in [31], which is defined as follows.

1. **Average**: For each program, the measurement *M *is calculated on each data set and then the usual arithmetic mean of the measurement *M *is evaluated for each program.

2. **Combined**: Adding up *nTP*, *nFP*, *nFN*, *nTN*, *sTP*, *sFP *and *sFN *over all data sets, the measurement *M *is computed for all data sets which are considered as a large data set.

3. **Normalized**: For each motif, the measurement *M *is normalized by subtracting the mean and dividing by the standard deviation over all the programs on that motif, and the average of these normalized scores over all motifs are obtained. This method puts easy and hard motifs on the same scale.

For finding TFBSs in the generated data sets from the above mentioned data bases, we use three test methods. In the first test method, for each TF, we have *t *known TFBSs of the length ℓ which are implanted in *t *sequences of the length *n*. Initially we calculate the motif matrix *B *and corresponding PWM. Now we scan *t *sequences with PWM and calculate the score of all subsequences of these *t *sequences based on three mentioned scoring schemas (independent scoring schema, dependent scoring schema, and our scoring schema) and then report subsequences with the score above a predefined *cutoff *(with value in the range of [0, 1]) as motif instances or predicted TFBSs. The above process is repeated for all TFs in each of the data sets. The value of *cutoff *is chosen based on best *nCC *for each TF. Roughly, one method for computing the *cutoff *is to fix an initial value for *cutoff *(rather a maximum value) and then select all subsequences of the length ℓ from *t *sequences with a score above this *cutoff*, as motif instances.

With regard to the known actual positions of binding sites each TF, the *nCC *value of this TFBSs is computed. Then we decrease the value of *cutoff *and we again predict TFBSs and calculate its corresponding *nCC*. If the value of *nCC *increases we repeat the whole process for smaller value of the *cutoff*, until we get to a *cutoff *whose *nCC *value decreases. The previous *cutoff *before this last decrease, is selected as the final *cutoff*. It should be noticed that, if *cutoff *is small, so *TP *and *FP *are large and *TN *and *FN *are small, and if *cutoff *is large then *TP *and *FP *are small and *TN *and *FN *are large. Therefore we choose *cutoff *such that the calculated *nCC *be maximum. In the second test method we use Jackknife method, again for each TF, we have *t *known TFBSs with the length ℓ which are implanted in *t *sequences with length *n*. First we ignore *j*-th TFBS of this set, then calculate the motif matrix *B *for *t *- 1 remaining TFBSs, and the corresponding PWM. Then based on the mentioned scoring schema, we consider *j*-th sequence and scan this sequence with PWM for finding a subsequence with maximum score as a predicted TFBS or motif instance. For each *j *(1 ≤ *j *≤ *t*) we repeat this process. Finally, accuracy of methods are investigated on all TFs in each data set. In the third test method, we use the method which is introduced in [24]. We have *m *= *t *+ *q *sequences of the length *n *and *t *TFBSs of the length ℓ for each TF which are implanted into *t *sequences of *m *sequences. Therefore *q *sequences have no motif. Now by using the value of *cutoff *which is calculated in the first test method for each TF, we try to find motif instances in *m *sequences by PWM of *t *known TFBSs. The accuracy of known motif prediction is investigated in all the above test methods for the predicted motifs.

Finally, we perform statistical analysis on *nCC *measurement of motifs in each data set (JASPAR, algorithm-Markov, algorithm-real, model-real, Generic, MChain and Real). In following we describe our statistical analysis. Let *P*_*i*, *j*, *k *_be the set of *nCC *values obtained from the *j*th test method (*1 *≤ *j *≤ 3) by the *k*th scoring schema (1 ≤ *k *≤ 3) on the *i*th data set (1 ≤ *i *≤ 7). Clearly *k *= 1, *k *= 2 and *k *= 3 stand for the independent, dependent and our scoring schema, respectively. Also *μ*_*i*, *j*, *k *_shows the average of the values given in the set *P*_*i*, *j*, *k*_. Now we intend to see whether the distribution of these values in the set *P*_*i*, *j*, *k *_follows a normal distribution or not. This is done by using the K-S (Kolmogorov-Smirnov) Test. Actually, this test represents whether the data shows a significant deviation from normality or not. Now if *p*-value is more than 0.05 thus the null hypothesis (*H*_0_), stating the data have come from normal distribution, is not rejected. We also compare the *nCC *values of our scoring schema with the two other scoring schemas (independent and dependent). For this reason, we compare the above mentioned mean values corresponding to each schemas. Mutually we compare (*μ*_*i*, *j*, 3_, *μ*_*i*, *j*, 1_) and (*μ*_*i*, *j*, 3_, *μ*_*i*, *j*, 2_) for each 1 ≤ *i *≤ 7 and 1 ≤ *j *≤ 3. For comparing *μ*_*i*, *j*, 3 _and *μ*_*i*, *j*, *k *_(*k *= 1 or *k *= 2), we use the paired t-test, if *P*_*i*, *j*, 3 _and *P*_*i*, *j*, *k *_have normal distribution, otherwise the Wilcoxon signed-ranks test is applied. This statistical analysis estimate significant deviation of two averages. The results of our statistical analysis are shown in the next subsections.

### JASPAR database

As mentioned, for extracting the data from JASPAR, all 107 TFs are selected from this database. Let us denote the number of TFBSs of the *i*th TF by *t*_*i*_, 1 ≤ *i *≤ 107. We implant TFBSs of each TF in some of random sequences that are extracted from the supplementary No. 8 enclosed in [24]. The number of these random sequences is 1800 and these sequences are of the length 250 to 500 and are sampled from a third-order Markov model background distribution. So we generate our data set as follows. Assume the *i*th TF in JASPAR, consists of *t*_*i *_TFBSs. We select randomly, *t*_*i *_sequences from 1800 background sequences and implant all these TFBSs in *t*_*i *_sequences in random position. We repeat this process for all TFs in JASPAR database. Finally 107 sets are obtained. Let *S*_*i *_be the set of *t*_*i *_sequences in which *t*_*i *_known TFBSs are implanted. The position dependency in this paper for evaluating dependent scoring schema is similar to the values given in [24], which are obtained by statistical approach with respect to their structures. Now, the performance of the above three test methods on generated data set are as follows. In the first test method, we use an ordinary search method. First, for the *i*th TF, the corresponding position weight matrix is constructed from its known TFBSs. Later, each subsequence *R *(|*R*| = ℓ) of *S*_*i *_is aligned to the constructed PWM and the *Score*_*j*_(*R*) and *NScore*_*j*_(*R*) (1 ≤ *j *≤ 3) are computed. Finally, the subsequences with the score above the *cutoff *are considered as motif instances or predicted TFBSs.

**Figure 1:**
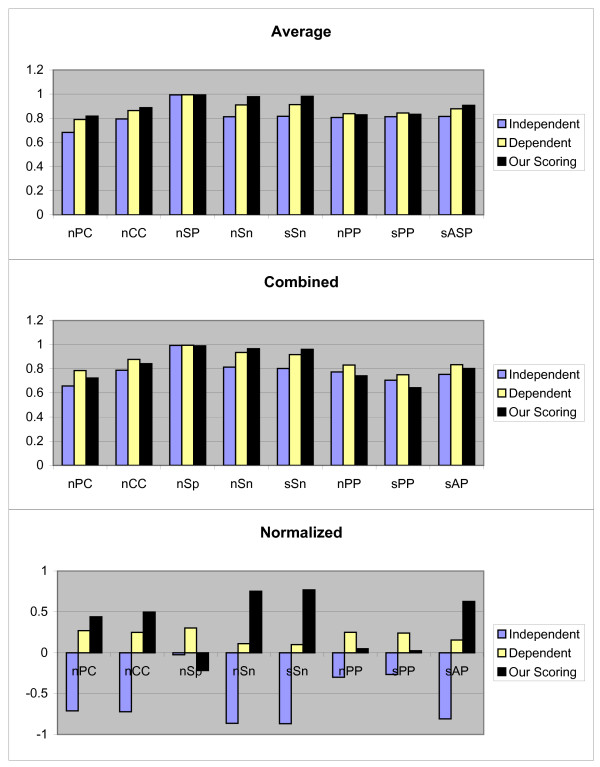
**Results obtained form three independent, dependent, and our scoring schemas, by the first test method on JASPAR data set**. These results include *nPC*, *nCC*, *nSp*, *nSn*, *nPP sPP*, and *sAP *values as shown in X axis. Y axis is numerically scaled based on 0.2 unit for these values. In this figure, results obtained by Average, Combined, and Normalized methods are shown from top to down respectively.

We again repeat the above process for all 107 TFs. Finally all previously mentioned measurements are evaluated. So for each measurement we obtain 107 values. For the obtained results the Average, Combined and Normalized results of each measurement (defined earlier) are shown in Figure 1. Although, our scoring schema is similar to dependent scoring schema but as seen in the figures our scoring schema can detect the motifs better. In following, we confirm this matter.

We have also performed some statistical analysis for 107 *nCC *values. For all the sets *P*_1, 1, *k*_, 1 ≤ *k *≤ 3, which do not follow a normal distribution, we use the Wilcoxon signed-ranks test on (*μ*_1, 1, 3_, *μ*_1, 1, 1_) and (*μ*_1, 1, 3_, *μ*_1, 1, 2_). The calculated *p*-values indicate that *μ*_1, 1, 3 _≥ *μ*_1, 1, 1 _(*p*-value = 1) and *μ*_1, 1, 3 _≥ *μ*_1, 1, 2 _(*p*-value = 0.8686).

**Figure 2:**
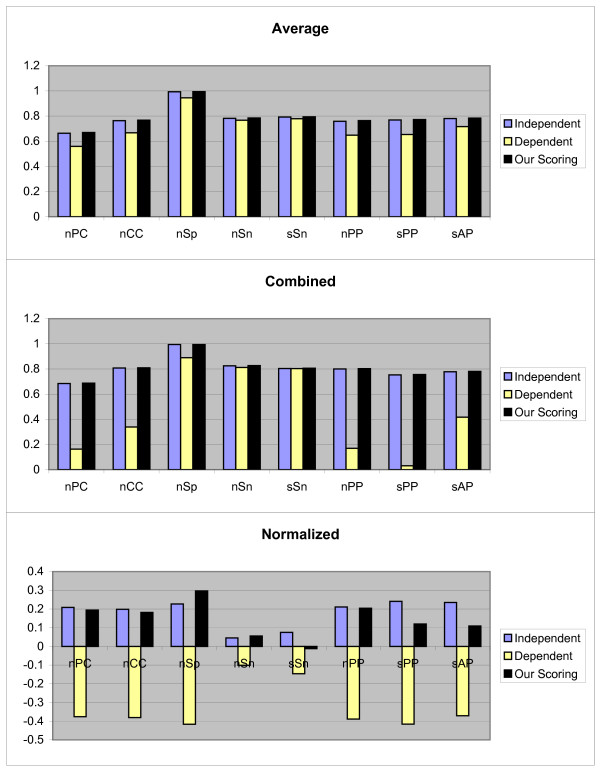
**Results obtained form three independent, dependent, and our scoring schemas, by the second test method on JASPAR data set**. These results include *nPC*, *nCC*, *nSp*, *nSn*, *nPP sPP*, and *sAP *values as shown in X axis. Y axis is numerically scaled based on 0.2 unit for these values. In this figure, results obtained by Average, Combined, and Normalized methods are shown from top to down respectively.

In the second test method, we use Jackknife method. Let us, assume that the *k*-th TF has *t*_*k *_TFBSs of the length ℓ, and *S*_*k *_be the set of sequences in which these *t*_*k *_TFBSs are implanted. Also, suppose that *j*-th TFBS is not known and *t*_*k *_- 1 TFBS are known. So by using the PWM of *t*_*k *_- 1 known TFBSs and the scoring schema, unknown *j*-th TFBS is predicted from *j*-th sequence in *S*_*i*_. For prediction, each subsequence *R *(|*R*| = ℓ) of the *j*th sequence is aligned with the constructed PWM and the *Score*_*i*_(*R*) and *NScore*_*i*_(*R*) (1 ≤ *i *≤ 3) are computed. The subsequences with maximum score are considered as motif instances. The above process is repeated for *j *= 1, 2,..., *t*, and *k *= 1, 2,..., 107, and all TFBSs with three scoring schemas are predicted. For these values the Average, Combined and Normalized results of each measurement are shown in Figure 2. In this case our scoring schema is similar to the independent scoring schema and is performed better than dependent scoring schema. In following, we again perform statistical analysis for confirming this result. We use the Wilcoxon signed-ranks test on (*μ*_1, 2, 3_, *μ*_1, 2, 1_) and (*μ*_1, 2, 3_, *μ*_1, 2, 2_). The calculated *p*-values indicate that *μ*_1, 2, 3 _≥ *μ*_1, 2, 1 _(*p*-value = 0.557) and *μ*_1, 2, 3 _≥ *μ*_1, 2, 2 _(*p*-value = 0.99971).

**Figure 3:**
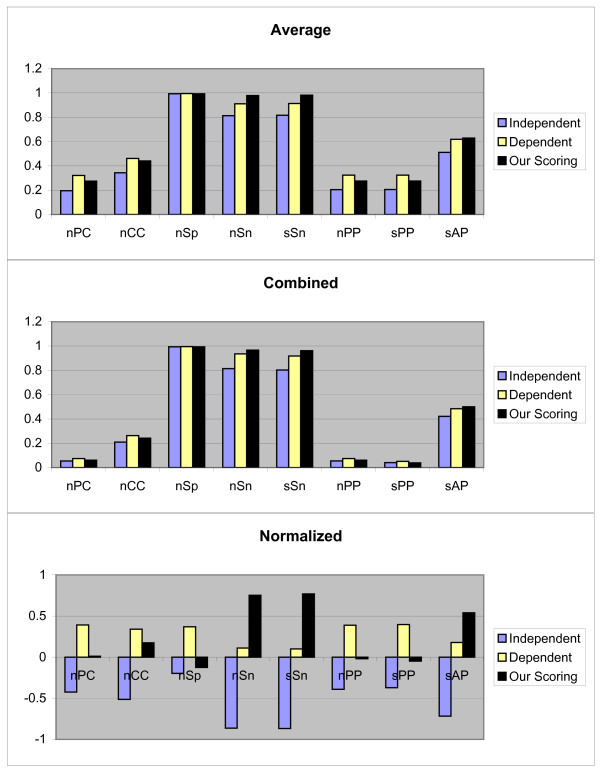
**Results obtained form three independent, dependent, and our scoring schemas, by the third test method on JASPAR data set**. These results include *nPC*, *nCC*, *nSp*, *nSn*, *nPP sPP*, and *sAP *values as shown in X axis. Y axis is numerically scaled based on 0.2 unit for these values. In this figure, results obtained by Average, Combined, and Normalized methods are shown from top to down respectively.

In the third test method, the previous 1800 true negative (TN) sequences (sequences without implanted motifs) are added to each 107 TFs. So the set *S*_*k *_of the *k*th TF has 1800 + *t*_*k *_sequences. Then the ability of each scoring schema for finding motifs for each TF is investigated. The employed search method is similar to the first test method. The Average, Combined and normalized results of each measurement in this test are shown in Figure 3. In this case our scoring schema and dependent scoring schema perform similarly. By notice that none of the sets *P*_1, 3, *k*_, 1 ≤ *k *≤ 3, follow a normal distribution, we use the Wilcoxon signed-ranks test on (*μ*_1, 3, 3_, *μ*_1, 3, 1_) and (*μ*_1, 3, 3_, *μ*_1, 3, 2_). The calculated *p*-values indicate that *μ*_1, 3, 3 _≥ *μ*_1, 3, 1 _(*p*-value = 0.9968) and *μ*_1, 3, 3 _≥ *μ*_1, 3, 2 _(*p*-value = 0.4696). We can see that our scoring schema is not case sensitive, but the performance of the other two scoring schemas are depend on test methods.

### Sandve's Benchmark

As mentioned, the data sets used for testing and comparing the three mentioned scoring schemas on TRANSFAC database, are the 'algorithm-Markov', 'algorithm-real', and 'model-real' bench mark data sets which are generated by Sandve et al. [30]. As mentioned in [30], these data sets are created by extracting the sets of binding site fragments with the same length for 213 different TF matrices. A binding site fragment is the binding site region that is used in the construction of a matrix in the TRANSFAC alignment. All three data set versions 'algorithm-Markov', 'algorithm-real', and 'model-real' are constructed from the same fragment sets and the selection of data sets is based on *nCC*. For the 'algorithm-real' version, binding sites are kept in their original genomic sequence, which is truncated to a maximum length of 2000 bp. To make the data sets more coherent, the binding site fragments that contained degenerate bases are removed. This binding sites have gaps in the TRANSFAC alignment, not located within the 2000 bp upstream of transcription start site in the sequence linked to by TRANSFAC. Additionally the selected motifs have *nCC *value higher than 0.79. For the 'algorithm-Markov' version, binding sites are implanted in the sequences generated from a third order Markov model inferred from all sequences of the corresponding real data set. In addition the selected motifs have *nCC *value higher than 0.87. Both the lengths of the 'algorithm-Markov' version sequences and the positions of the implanted binding sites are kept equal to the corresponding real sequences. Motifs with fewer than five binding sites are removed, and 50 motifs (each motif has some motif instances or TFBSs) for 'algorithm-real' and 50 motifs for 'algorithm-Markov' are kept. For creating 'model-real' version, 25 motifs with *nCC *below 0.72 are selected. Each of these motifs have at least 18 motif instances (bing sites) and are kept in their original genomic sequences. It should be noted that in each motif, motif instances are similar to the background sequences (*nCC *≤ 0.72).

**Figure 4:**
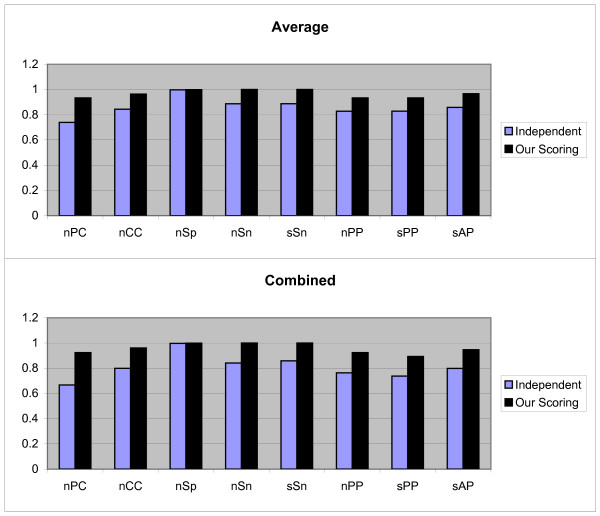
**Results obtained form two independent and our scoring schemas, by the first test method on 'algorithm-Markov' sandve's benchmark**. These results include *nPC*, *nCC*, *n*Sp, *n*Sn, *n*PP *s*PP, and sAP values as shown in X axis. Y axis is numerically scaled based on 0.2 unit for these values. In this figure, results obtained by Average and Combined methods are shown from top to down respectively.

**Figure 5:**
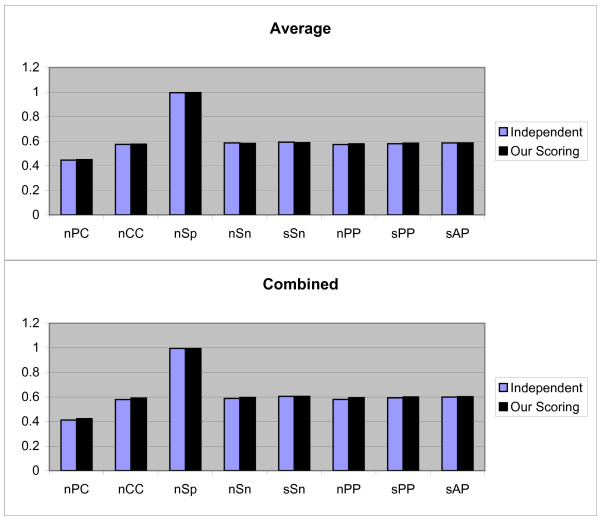
**Results obtained form two independent and our scoring schemas, by the second test method on 'algorithm-Markov' sandve's benchmark**. These results include *nPC*, *nCC*, *nSp*, *nSn*, *nPP sPP*, and *sAP *values as shown in X axis. Y axis is numerically scaled based on 0.2 unit for these values. In this figure, results obtained by Average and Combined methods are shown from top to down respectively.

We have run the benchmark data sets with both independent position scoring and our scoring schema, but not with dependent scoring schema; since dependency between the positions of motifs are not available for these data sets, therefore the dependent scoring schema can not be tested on these data sets. For each data set in this benchmark, the test have been done by the first and second test methods discussed in above and the Average and Combined results of each measurement are obtained. The third testing method is not implemented, because this benchmark has no information about TN sequences (sequences that do not contain any motifs) and we would like to keep the originality of this benchmark. The Figures 4 and 5 show the results of first and second test methods on 'algorithm-Markov' data sets respectively (note that, in this test the Normalized method is not employed because this method is not good when we have two cases).

We have also perform statistical analysis on 50 *nCC *values on the first and second test methods. Since *P*_2, 1, 1 _and *P*_2, 1, 3 _which do not follow normal distribution, we use the Wilcoxon signed-ranks test on (*μ*_2, 1, 3_, *μ*_2, 1, 1_) do not follow *P*_2, 2, 1 _and *P*_2, 2, 3 _do not follow distribution. The calculated *p*-values indicate that *μ*_2, 1, 3 _≥ *μ*_2, 1, 1 _(*p*-value = 1) and *μ*_2, 2, 3 _≥ *μ*_2, 2, 1 _(*p*-value = 0.5316).

**Figure 6:**
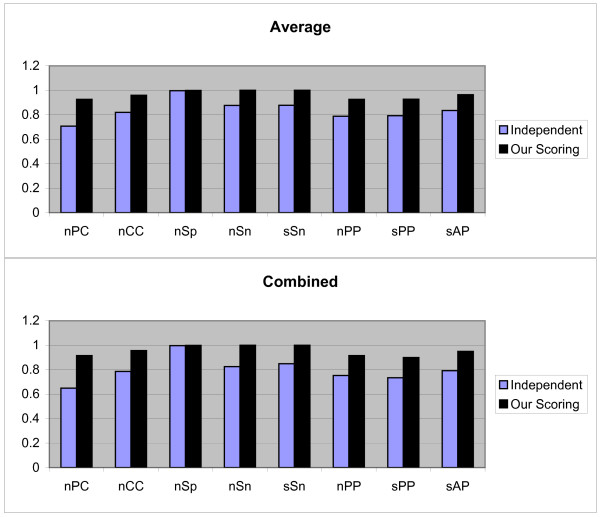
**Results obtained form two independent and our scoring schemas, by the first test method on 'algorithm-real' sandve's benchmark**. These results include *nPC*, *nCC*, *nSp*, *nSn*, *nPP sPP*, and *sAP *values as shown in X axis. Y axis is numerically scaled based on 0.2 unit for these values. In this figure, results obtained by Average and Combined methods are shown from top to down respectively.

**Figure 7:**
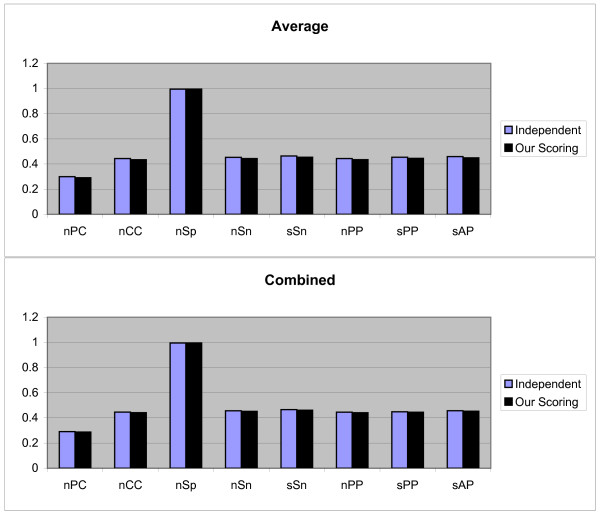
**Results obtained form two independent and our scoring schemas, by the second test method on 'algorithm-real' sandve's benchmark**. These results include *nPC*, *nCC*, *nSp*, *nSn*, *nPP sPP*, and *sAP *values as shown in X axis. Y axis is numerically scaled based on 0.2 unit for these values. In this figure, results obtained by Average and Combined methods are shown from top to down respectively.

Also, the Figures 6 and 7 show the results first and second test methods on 'algorithm-real' data sets respectively. We have also done statistical analysis on 50 *nCC *values on the first and second test methods. Since *P*_3, 1, 1 _and *P*_3, 1, 3 _which do not follow normal distribution, we use the Wilcoxon signed-ranks test on (*μ*_3, 1, 3_, *μ*_3, 1, 1_) but *P*_3, 2, 1 _and *P*_3, 2, 3 _follow normal distribution therefore we use the t-test on (*μ*_3, 2, 3_, *μ*_3, 2, 1_). The calculated *p*-values indicate that *μ*_3, 1, 3 _≥ *μ*_3, 1, 1 _(*p*-value = 1) and *μ*_3, 2, 3 _≥ *μ*_3, 2, 1 _(*p*-value = 0.7774).

**Figure 8:**
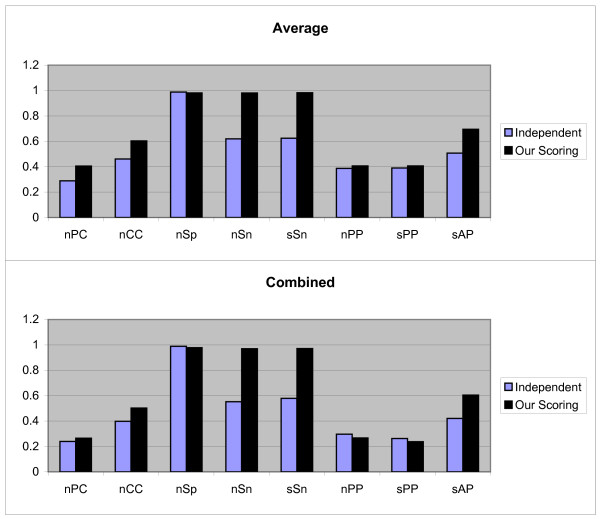
**Results obtained form two independent and our scoring schemas, by the first test method on 'model-real' sandve's benchmark**. These results include *nPC*, *nCC*, *nSp*, *nSn*, *nPP sPP*, and *sAP *values as shown in X axis. Y axis is numerically scaled based on 0.2 unit for these values. In this figure, results obtained by Average and Combined methods are shown from top to down respectively.

**Figure 9:**
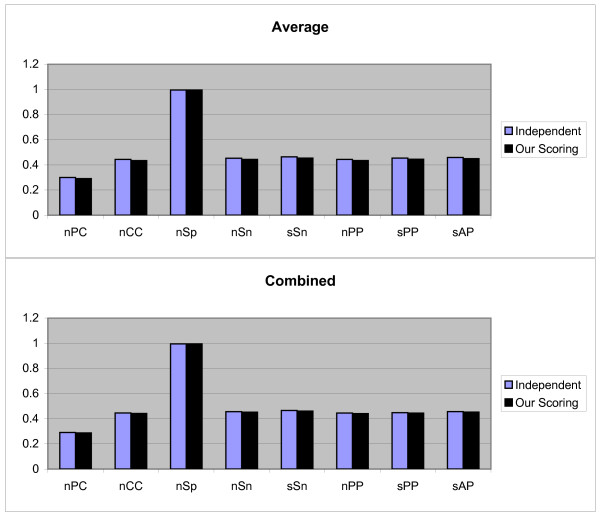
**Results obtained form two independent and our scoring schemas, by the second test method on 'model-real' sandve's benchmark**. These results include *nPC*, *nCC*, *nSp*, *nSn*, *nPP sPP*, and *sAP *values as shown in X axis. Y axis is numerically scaled based on 0.2 unit for these values. In this figure, results obtained by Average and Combined methods are shown from top to down respectively.

The Figures 8 and 9 show the results of first and second test methods on 'model-real' data sets respectively. We have also perform statistical analysis on 25 *nCC *values on the first and second test methods. Since *P*_4, 1, 1 _and *P*_4, 1, 3 _which follow normal distribution, we use the t-test on (*μ*_4, 1, 3_, *μ*_4, 1, 1_) and so *P*_4, 2, 1 _and *P*_4, 2, 3 _are normal distribution. The calculated *p*-values indicate that *μ*_4, 1, 3 _≥ *μ*_4, 1, 1 _(*p*-value = 1) and *μ*_4, 2, 3 _≥ *μ*_4, 2, 1 _(*p*-value = 0.2818).

As we can see, in Jackknife testing method, our scoring schema and independent scoring schema are similar, but our scoring schema performs better when the motif instances are planted in the data sets and the search method is based on *cutoff*.

### Tompa's benchmark

As mentioned, other data sets used for testing and comparing the two mentioned scoring schemas on TRANSFAC data base, are the 'Generic', 'MChain', and 'Real' data sets which are generated by Tompa et al. [31]. Recall from [31], the data set 'Real' is created by implanting the selected TFBSs of TRANSFAC into real promoter sequences as a background, the data sets 'Generic' are created by implanting the selected TFBSs of TRANSFAC into randomly chosen promoter sequences from the same genome, and the data set 'MChain' is created by implanting the selected TFBSs of TRANSFAC into sequences generated by a Markov chain of order 3. The implanted TFBSs do not have the same length in all three data set types. The TFBSs are selected from TRANSFAC by the following process. Initially, only TFs are selected for which TRANSFAC also lists a binding site consensus sequences. For each factor, duplicate instances of the same binding site, binding sites missing sequence or position information, binding sites whose position is annotated as start site, binding sites whose position is less than -3000 or greater than 0, and sequences with two reported binding sites contradicting each other in the sequence are removed. The remaining binding sites are implanted into three type of background sequences. In addition in each data set some sequences without motifs are also inserted and consequently 52 motifs of each type are obtained. Since each data set contains some motif with non-similar length, and some sequences in each data sets do not have any motif, we omit some sequences in the data sets and finally, for each data set types, we have chosen 16 motifs that at least have 4 motif instances with the same length. It should be noted that the PWM is made from this data but the test is done on all member of these data set.

**Figure 10:**
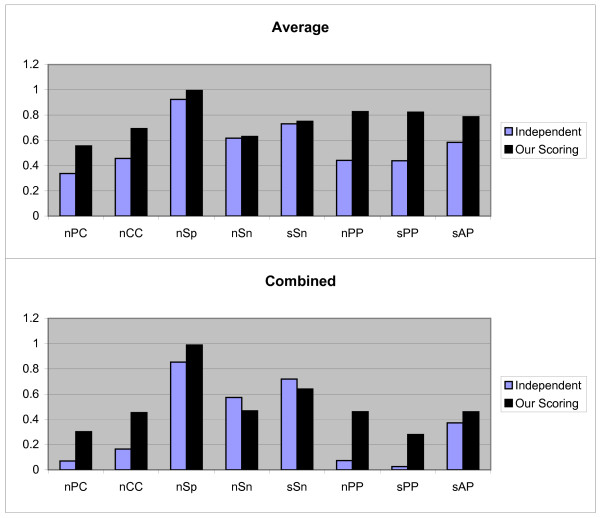
**Results obtained form two independent and our scoring schemas, by the third test method on 'Generic' Tompa's benchmark**. These results include *nPC*, *nCC*, *nSp*, *nSn*, *nPP sPP*, and *sAP *values as shown in X axis. Y axis is numerically scaled based on 0.2 unit for these values. In this figure, results obtained by Average and Combined methods are shown from top to down respectively.

**Figure 11:**
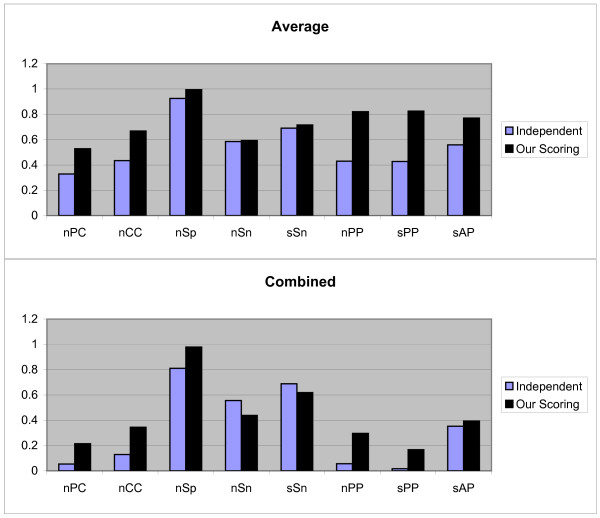
**Results obtained form two independent and our scoring schemas, by the third test method on 'MChain' Tompa's benchmark**. These results include *nPC*, *nCC*, *nSp*, *nSn*, *nPP sPP*, and *sAP *values as shown in X axis. Y axis is numerically scaled based on 0.2 unit for these values. In this figure, results obtained by Average and Combined methods are shown from top to down respectively.

**Figure 12:**
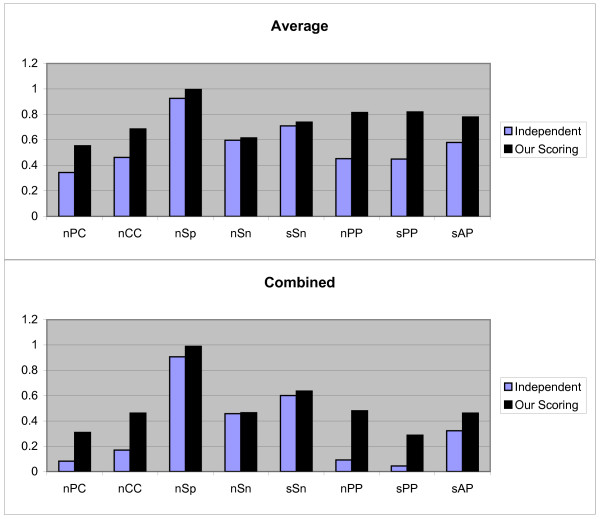
**Results obtained form two independent and our scoring schemas, by the third test method on 'Real' Tompa's benchmark**. These results include *nPC*, *nCC*, *nSp*, *nSn*, *nPP sPP*, and *sAP *values as shown in X axis. Y axis is numerically scaled based on 0.2 unit for these values. In this figure, results obtained by Average and Combined methods are shown from top to down respectively.

Similar to the Sandve's benchmark data sets we have run this benchmark with independent position scoring and our scoring schemas, but not with dependent scoring schema; since dependency between the positions of motifs are not available for these data sets, therefore the dependent scoring schema can not be tested on these data sets. For each samples in this benchmark, third test method have been done. First, the PWM corresponding to motifs in each data set are constructed, and then we suppose these motif are known and we try to predict motifs in all data set background sequences. The Average and Combined obtained results of each measurement on this benchmark based on 'generic', 'Markov', and 'Real' data sets are shown in Figures 10, 11, and 12 respectively. We have also done statistical analysis on 16 *nCC *values on the first and second test methods. *P*_5, 3, 1 _and *P*_5, 3, 3 _which follow normal distribution so we use t-test on (*μ*_5, 3, 1_, *μ*_5, 3, 3_). The calculated *p*-values indicate that *μ*_5, 3, 3 _≥ *μ*_5, 3, 1 _(*p*-value = 1). *P*_6, 3, 1 _and *P*_6, 3, 3 _which do not follow normal distribution, we use the Wilcoxon signed-ranks on (*μ*_6, 3, 1_, *μ*_6, 3, 3_). The calculated *p*-value indicates that *μ*_6, 3, 3 _≥ *μ*_6, 3, 1 _(*p*-value = 0.9881). *P*_7, 3, 1 _and *P*_7, 3, 3 _which follow normal distribution so we use the t-test on (*μ*_7, 3, 1_, *μ*_7, 3, 3_). The calculated *p*-value indicates that *μ*_7, 3, 3 _≥ *μ*_7, 3, 1 _(*p*-value = 0.9843). In this case we can also see that our scoring schema is performed better than independent scoring schema in each data set.

## Conclusion

In this work, we investigate the dependencies within transcription factor binding sites, and present a simple way for modeling these dependencies. We have developed a new scoring schema for known binding site perdition. In this scoring schema the joint information content and mutual information are used as a measure of dependency between position in TFBS. We have evaluated different aspects of the scoring schema and this method is implemented and tested on real data sets. The results are compared with two well known scoring schemas. For comparison some statistical measurements are considered which show our scoring schema can improve motif prediction.

For investigating the improvement of our scoring schema on sTP, we calculated sTP of three scoring schema on all seven tested data sets and we observed that, on all the tested data sets our scoring schema show an average %21 improvement comparing to the independent scoring schema and also %11 improvement comparing to the dependent scoring schema on *sTP*.

For indicating the predicting power of our approach against the independent scoring schema, we have performed a gene wide search on Yeast genome which consists of 16 chromosomes, for REB1 TF with 19 TFBSs, ROX1 TF with 8 TFBSs, UASH TF with 21 TFBSs and URS1 TF with 14 TFBSs [33]. For each TF, a profile is created based on its TFBSs, and each TFBS is scored by its profile based on our scoring schema. The minimum obtained score is considered as a *cutoff *for this TF for our scoring schema. With respect to this *cutoff *value, the Yeast genome is searched for detecting these TFBSs with our scoring schema, and all subsequences with a score above the *cutoff *are reported as motif instances. Finally the nTP, nFN, and nFP criteria are calculated for these motifs. The above process is also repeated for independent scoring schema and the nTP, nFN, and nFP criteria for motif instances which found by this scoring schema, are also calculated. The Table 1 show the nTP, nFN, and nFP values obtained by our scoring schema and independent scoring schema for detecting TFBSs of REB1, ROX1, UASH and URS1. As we can see, in this table values of these criteria of our scoring schema are higher, which show a better prediction.

**Table 1:** The nTP, nFN, and nFP values obtained by our scoring and independent scoring schema on REB1, ROX1, UASH and URS1.

Scoring Schema	TF	*nTP*	*nFN*	*nFP*
Our scoring	REB1	140	64	16590

Independent scoring	REB1	140	64	7264880

Our scoring	ROX1	96	8	1632

Independent scoring	ROX1	96	8	5067288

Our scoring	UASH	270	66	2130

Independent scoring	UASH	270	66	347040

Our scoring	URS1	182	0	1534

Independent scoring	URS1	182	0	115427

In general, the obtained results on the biological data sets demonstrated that the joint information content and mutual information provide a better and more general criterion to investigate the relationship between positions in the TFBS, and motif detection can be improved with the scoring schema that considers dependency in TFBSs.

## Authors' contributions

Initial idea of the research was from FZM and MS. All authors participated in designing the structure and organization of the manuscript. FZM designed and implemented the scoring schema and tested on different data sets. All authors contributed to read and approved the final manuscript.
